# Conceptual definition for drowning prevention: a Delphi study

**DOI:** 10.1136/ip-2023-045085

**Published:** 2023-11-09

**Authors:** Justin-Paul Scarr, Jagnoor Jagnoor

**Affiliations:** 1 Injury Division, The George Institute for Global Health, University of New South Wales, Sydney, New South Wales, Australia; 2 Royal Life Saving Society - Australia, Broadway, New South Wales, Australia; 3 Injury Division, The George Institute for Global Health, New Delhi, India

**Keywords:** Drowning, Public Health, Policy

## Abstract

**Background:**

Expanding support for drowning prevention is evidenced by interlinked Resolutions at the United Nations (2021) and World Health Assembly (2023). While progress has accelerated, a universally agreed definition for drowning prevention remains absent. Here, we aim to develop a conceptual definition of drowning prevention using the Delphi method.

**Methods:**

First, we conducted a document review to guide our development and consensus-building process. Then, we formed an advisory group and recruited participants with diverse expertise to contribute to Delphi-method surveys. In the first round, participants selected from draft concepts to build a definition and delineate between the terms drowning prevention and water safety. In the second round, we presented a codeveloped definition, and three statements based on first-round findings. We then sought participant feedback where ≥70% support was considered consensus-based agreement.

**Results:**

Participants (n=134) were drawn from community (7.46%), policy (26.87%), research (40.30%) and technical backgrounds (25.37%), and low-income and middle-income countries (38.06%). In the first- round, half (50.74%) disagreed with the proposition that drowning prevention was synonymous to water safety, while 40.30% agreed. The second- round achieved consensus-based agreement (97.27%) for the definition: Drowning prevention is defined as a multidisciplinary approach that reduces drowning risk and builds resilience by implementing evidence-informed measures that address hazards, exposures and vulnerabilities to protect an individual, community or population against fatal and non-fatal drowning.

**Conclusion:**

The Delphi method enabled the codevelopment of our conceptual definition for drowning prevention. Agreement on the definition forms the basis for strengthened multisectoral action, and partnerships with health and sustainable development agendas. Defining drowning prevention in terms of vulnerability and exposure might increase focus on social determinants and other upstream factors critical to prevention efforts.

**WHAT IS ALREADY KNOWN ON THIS TOPIC**

- Drowning prevention has experienced increasing prominence in global health and sustainable development discourse in recent years.
- Studies reinforce the importance of framing drowning and drowning prevention in ways to inspire action, expand coalitions and strengthen institutions for multisectoral action.

**WHAT THIS STUDY ADDS**

- Our study presents a consensus-based conceptual definition for drowning prevention, including the key concepts of risk, hazard, exposure, vulnerability and resilience.
- Agreement on the definition for drowning prevention forms the basis for strengthened multisectoral action, and partnerships with health and sustainable development agendas.

**HOW THIS STUDY MIGHT AFFECT RESEARCH, PRACTICE OR POLICY**

- Defining drowning prevention in terms of vulnerability and exposure might increase focus on social determinants and other upstream factors critical to prevention efforts.
- The definition forms the basis for further investigation of how sectors and agendas not yet engaged in drowning prevention might identify cobenefits and work to codevelop interventions in ways not previously considered.

## Introduction

Drowning is a complex, and often overlooked public health issue, where global mortality estimates exceed 236 000 deaths annually, excluding drowning in disaster and transportation.^1 2^ Methodological advancements in population-representative data, growing evidence for effective interventions and an elevated focus on high burdens in low-income and middle-income countries (LMICs), have improved the visibility of the problem and coalesced action for drowning prevention.^3^

Drowning prevention’s rise in health and sustainable development discourse is evidenced by successive Resolutions, first at the United Nations General Assembly (2021),^4^ and then at the World Health Assembly (2023).^5^ Significant increases in drowning research,^6^ and the publication of reports by WHO,^7–9^ reinforce further growth. Yet, there is no universally agreed definition for drowning prevention.^10^

Significant ambiguity in the conceptualisations of drowning prevention across peer-reviewed and grey literature has been observed.^6 10^ The terms drowning prevention and water safety are used interchangeably or with different or overlapping meanings.^10^ Discordance around terminologies and a lack of consensus-based agreement for key terms, has the potential to hinder cohesive progress.^3 6^ Reaching a consensus on the definition of drowning prevention has the potential to advance the research agenda,^11^ foster stronger partnerships with stakeholders in other health and sustainable development initiatives,^10^ and facilitate the pursuit of strategic priorities through the formulation of a global strategy.^12^

Previous consensus-based processes have had significant benefits for advancing drowning prevention.^3^ For example, the development of a standardised definition for drowning in 2002,^13^ was adopted by WHO and led to increased consistency in reporting of the drowning burden.^14^ The Delphi method has been used to form consensus-based agreement on preventative messaging in recreational drowning prevention settings,^15^ and to develop agreement on prevention strategies for drowning in rivers.^16^

Here, we aim to develop a conceptual definition for drowning prevention and reach consensus-based agreement for its adoption using the Delphi method. We seek to develop shared terminology to strengthen multisectoral action for drowning prevention.

## Methods

### Study design

**Figure 1:**
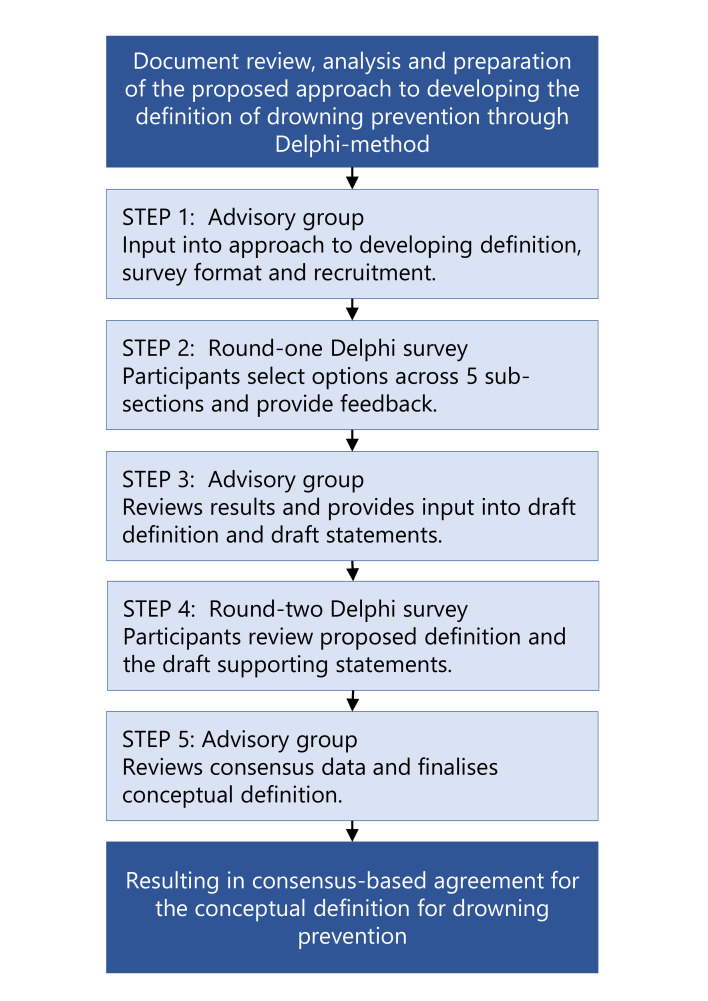
Five-step Delphi consensus-based agreement process.

We applied the Delphi method in a five-step process (figure 1). The Delphi method is a well-established approach to forming a consensus-based agreement across subject matter experts. The Delphi method has been applied to create conceptual definitions for major trauma,^17^ advance care planning^18^ and running-related injury.^19^

### Advisory group

We formed an advisory group (n=6) by recruiting members from the existing networks of both authors. Advisory group members held expertise in research, policy and practice, and had experience in a breadth of drowning prevention contexts including LMICs. The advisory group met twice (virtually) to provide input into study design, participant recruitment, survey development and analysis of results. The advisory group established that consensus-based agreement would be ≥70% of participants agreeing to the final draft definition or the proposed statements.

### Participant recruitment

Participants were identified through document analysis, from the existing research, policy and practitioner networks of both authors, and referrals from the advisory group. International and national drowning prevention groups including International Life Saving Federation, Royal Life Saving Society Commonwealth, and Royal Life Saving Society—Australia, as well as The George Institute for Global Health were engaged to promote the study by posting advertisements on their social media accounts, or by sending an email invitation to potential participants.

The inclusion criteria were based on expertise: policy makers, researchers, technical specialists or community members, and work setting: UN agencies, governments, non-government organisations, foundations, academic institutions or from communities affected.

Potential participants reviewed an online information and consent form. When participants enrolled in the study and provided consent, they were sent a link to the first-round Delphi survey, which we deployed using online software REDCap.^20^

### Preparation: identifying key concepts for inclusion

We used document review of peer-review and grey literature to identify concepts used to describe drowning prevention. The search of both peer-review and grey literature aimed to capture contexts and terminology for drowning prevention across research, policy and practice.

First, we searched four databases: MEDLINE (via PubMed), The Cochrane Library, Web of Science and Embase for peer review literature using the search terms “drowning,” “drowning MESH” and “near drowning” in titles, abstracts and keywords for the period 1 January 2005 to 31 December 2020.

Then, we searched four databases: WHO library- Iris, UNICEF website database, Google, Relief Web for grey literature, including global, regional, or national-level reports and planning documents using the search terms “drowning” and “drowning and water safety” for the period 1 January 2014 and 31 December 2020. The WHO library-Iris, UNICEF and Relief Web were chosen to capture grey documents in the UN system, and various sustainable development agendas. The search method and data extraction is reported in our scoping review that identified opportunities for multisectoral action for drowning prevention.^10^

We conducted content analysis, by first extracting descriptive text blocks from the literature and grey documents where the term ‘drowning prevention’ was used. We then assigned each block of text a code, and then categorised the codes into frames. Frames are defined as the central idea being presented in the text.^21^ The thematically extracted frames informed the initial approach to drafting the subsections of potential draft definition. These subsections and the overall approach were reviewed by both authors and the advisory group, before the drafts were again discussed and confirmed in preparation for the first-round.

### First-round: participant selection of concepts for inclusion

We designed the first-round survey in two parts. In part one, we proposed five sections for a potential definition. Once participants made their choices for each section, their choices were combined and displayed in draft definition format to allow for further consideration (online supplemental file 1).

10.1136/ip-2023-045085.supp1 Supplementary data

In part two of the survey, we assessed participant views on the relationship between drowning prevention and water safety. Participants were presented with the statement—drowning prevention and water safety are synonyms and can be used interchangeably and asked to indicate their view on a five-point Likert scale (strongly agree—agree—neutral—disagree—strongly disagree). Open text fields were provided to collect feedback for both parts.

### Second-round: building consensus-based agreement

In the second-round Delphi survey, we presented a draft conceptual definition, which was codeveloped using the highest scoring concepts from each section in the first-round survey, and after considering participant and advisory group feedback. We presented three statements aiming to address the feedback collected about confusion between the terms drowning prevention and water safety.

### Patient and public involvement statement

There was no public/patient involvement in this study. Participants will receive a link to access the results.

### Role of the funding source

The funding source (Australian Government Research Training Programme Scholarship) played no role in the study.

## Results

### Delphi participants

We recruited 134 experts from 46 countries. Participants were drawn from community (n=10, 7.46%), policy (n=36, 26.87%), research (n=54, 40.30%) and technical backgrounds (n=34, 25.37%), and LMICs (n=51, 38.06%) (table 1).

**Table 1:** Characteristics of Delphi participants

Participant data	First round		Second round	
	n	%	n	%
Participants	134	100	110	82.09
Countries	46	100	40	86.96
Area of expertise for participants				
Community based	10	7.46	6	5.45
Policy or planning	36	26.87	29	26.36
Research	54	40.30	49	44.55
Technical services	34	25.37	26	23.64
Economic categories of countries				
High-income country	83	61.94	74	67.27
Low-income and middle-income country	51	38.06	36	32.73
WHO regions representation				
Africa region	18	13.43	12	10.91
Americas region	26	19.41	23	20.91
Eastern Mediterranean region	3	2.24	3	2.73
Europe region	34	25.37	29	26.36
South-East Asia region	23	17.16	17	15.45
Western Pacific region	30	22.39	26	23.64

The highest representation came from WHO Europe region (n=34, 25.37%) and the lowest was from the WHO Eastern Mediterranean region (n=3, 2.24%). The second-round survey achieved a response rate of 82.09% (n=110).

### Conceptual framework for the definition

We developed a conceptual framework consisting of five sections for the definition. (1) Our document review determined that drowning prevention in the literature is either used as a concept, a practice, a multidisciplinary or a public health approach, or as an action. (2) Drowning prevention aims included to reduce risk, build resilience, implement measures, or address hazards, exposures, and vulnerability. (3) The focus of drowning prevention ranged from a person to people, or a combination of individuals, communities and populations. (4) Studies commonly used the terms submersion, fatal and non-fatal submersion, and sometimes included other injuries. (5) Terminologies included a body of water, any liquid, or ‘in, on or around a body of water’. These concepts were organised into sections and presented to participants as options in the first-round Delphi survey. Following discussion with the advisory group, the research team developed descriptors for key concepts presented within the options (table 2).

**Table 2:** Descriptors for drowning risk, hazard, vulnerability and exposure

Concept	Descriptor	Examples
Drowning risk	Drowning risk is the consequence of the interaction between a hazard and the characteristics that make people vulnerable to drowning and their exposure (context, frequency of occurrence, duration) to a drowning hazard.	See below
Drowning hazard	A drowning hazard is an environmental or human induced feature or process that contributes to drowning risk.	Environmental features include oceans, rivers, lakes and beaches. Environmental processes include tropical storms, floods, tsunami, heatwaves, currents, waves steep or slippery banks Human induced processes and features in the built environment or flaws in technological design, for example, modes of transportation, swimming pools, bathtubs and structurally deficient dams.
Drowning exposure	Drowning exposure is the context, frequency of occurrence and duration for which an individual, community or population interacts with a specific drowning hazard.	Exposure can be influenced by social, economic and environmental factors, including occupational, recreational or everyday living contexts.
Drowning vulnerability	Drowning vulnerability is the susceptibility to drowning of an individual, a community or population.	Vulnerability is best measured in layers and can be based on a range of physical, social, economic and environmental factors. For example, age, residential status, access to education and health services.
Drowning resilience	Drowning resilience is the adaptive and robust capacity of an individual, community or system to avoid, reduce or respond to drowning risk.	Individual-level resilience might include the development of swimming and basic rescue skills. Community-level resilience might include the availability of safe places to swim, or the presence of a lifeguard service. System-level resilience might include pool fencing legislation, safety standards or a code of practice for waterways and swimming pools, or enforced lifejacket laws.

### First-round results

#### Selection of concepts for inclusion

The highest rated response options were (1) a multidisciplinary approach (n=83, 61.94%), (2) addressing drowning hazards, exposures and vulnerabilities (n=95, 70.90%), (3) protecting an individual, community or population (n=119, 88.81%), (4) against fatal and non-fatal submersion, and other injuries (n=63, 47.02%) and (5) in, on or around a body of water (n=82, 61.19%) (table 3). Suggested adjustments included to using drowning instead of submersion and replacing ‘a body of water’ with ‘water’.

**Table 3:** First-round results: participant (n=134) selection of concepts for inclusion in the definition

Component of definition	n	%
(1) Drowning prevention is		
a concept or practice	3	2.24
a multidisciplinary approach	83	61.94
a public health approach	21	15.67
an approach	6	4.48
any action	21	15.67
(2) That aims to (multiple choices allowed)		
reduce risk and build resilience	73	54.48
address drowning hazards, exposures and vulnerabilities	95	70.90
implement measures	69	51.49
(3) To protect		
a person	2	1.49
an individual, community or population	119	88.81
people	13	9.70
(4) Against		
drowning	46	34.33
fatal and non-fatal submersion	23	17.16
fatal and non-fatal submersion, and other injuries	63	47.02
submersion	2	1.49
(5) In		
a body of water	29	21.64
a liquid	23	17.1
In, on or around a body of water	82	61.19

The section titled ‘that aims to’ allowed for multiple responses. Here the top-rating choice was addressing drowning hazards, exposures and vulnerabilities (n=95/134, 70.90%). After advisory group consultation, reduce risk and build resilience (n=72/134, 54.48%) and implement measures (n=69/134, 51.49%) were added to the draft definition as they were rated highly by ≥50% of participants.

#### Drowning prevention and water safety are synonyms, or not?

When presented with the statement—drowning prevention and water safety are synonyms and can be used interchangeably, participants either agreed and strongly agreed (n=54, 40.30%), disagreed and strongly disagreed (n=71, 50.74%) or were neutral (n=12, 8.96%). (Data not shown)

Open field feedback in response to the above question (n=50, 37.31%) exhibited overlapping characteristics, regardless of whether participants agreed or disagreed on that drowning prevention and water safety were synonyms. Themes included, (1) drowning prevention is a more focused concept (n=22, 44.00%), (2) drowning prevention involves more complex system-based approaches than those used in water safety (n=10, 10.00%) and (3) using drowning prevention avoids confusion with safe drinking water, sanitation and hygiene (WASH) programmes (n=13, 26.00%) (table 4).

**Table 4:** First-round: themes addressing differences between drowning prevention and water safety

Theme	Participant feedback example
Focus—Drowning prevention is a more focused term, and it is a subset of water safety	*While sometimes used interchangeably, water safety is a broader concept in my opinion. It includes all injuries that can occur on or around an aquatic setting and can include diseases regarding water quality too. Drowning prevention is part of the water safety concept.* Respondent 21, research expert
	*Water safety is promoting safe behaviours in on and around water whereas drowning prevention focuses solely on drowning*. Respondent 107, technical expert
	*I understand many (high-income) governments favour the term as it implies the agenda is broader than 'drowning prevention' in the classic sense*. Respondent 17, policy expert
Complexity—Drowning prevention involves more complex systematic approaches	*Water safety refers to measures taken to ensure that individuals are safe in or around water, including the use of personal flotation devices, swimming lessons, and pool rules. Drowning prevention, on the other hand, specifically focuses on reducing the risk of drowning. This may include water safety measures, but also encompasses other strategies such as drowning response and rescue, drowning risk assessment, and drowning surveillance and research.* Respondent 61, policy expert
	*Water safety is a specific set of strategies/actions to target individuals in their interaction with aquatic environments, whereas drowning prevention may include some specific actions, though is a much more holistic approach to consider the environmental impacts, the public efforts, geographical situation, economics, and resource implications.* Respondent 47, research expert
Context—Using drowning prevention can avoid confusion with safe drinking water and sanitation, hygiene (WASH)	*The term water safety can be confused with ’safe drinking water' depending on the context in which the term water safety is used. I suggest that when using the term ‘water safety’ it should include an additional term/explanatory to separate this from ’safe drinking water'. Especially when considering a multidisciplinary approach with WASH sectors.* Respondent 72, policy expert
	*Within the UN system, ‘water safety’ is strongly linked to the microbiological safety of drinking water, which is linked to the WASH agenda. As a result, I avoid using the term 'water safety' when speaking to UN colleagues.* Respondent 17, research expert
	*Water safety is a broad term that is also used in sanitation approaches and so using it interchangeably with drowning prevention is misleading.* Respondent 106, research expert

Quotes are in *italics*.

UN, United Nations; WASH, Water and sanitation, hygiene.

### Second-round results

#### High-level of support for the conceptual definition

The conceptual definition presented in the second-round survey was supported by 97.27% of participants (n=107), exceeding the consensus-based criteria of ≥70% participant agreement. Three participants did not support the proposed definition (n=3, 2.72%). Analysis of their open text feedback (n=2) indicated a belief that the proposed definition was too complex or that a moderately refined version was preferred.

Therefore, our consensus-based conceptual definition is:

Drowning prevention is defined as a multidisciplinary approach that reduces drowning risk and builds resilience by implementing evidence-informed measures that address hazards, exposures, and vulnerabilities to protect an individual, community or population against fatal and non-fatal drowning.

#### High-level of support for the statements

We presented three statements to strengthen the definition, and address the themes (focus, complexity, context) identified in the first-round survey. All supporting statements achieved a high level of consensus-based agreement (table 5).

**Table 5:** Consensus definition and supporting statements

Section	Detail
Definition	Drowning prevention is defined as a multidisciplinary approach that reduces drowning risk and builds resilience by implementing evidence-informed measures that address hazards, exposures and vulnerabilities to protect an individual, community or population against fatal and non-fatal drowning.
Statement 1	While drowning prevention is not a synonym for water safety, both approaches are closely related and can share measures, and address common hazards, exposures, and vulnerabilities.
Statement 2	When combined, drowning prevention and water safety, includes measures that aim to promote safety and prevent injuries and other incidents in, on and around water.
Statement 3	Using 'drowning prevention' when engaging with sectors where water safety is principally associated with safe drinking water, sanitation and hygiene' can help reduce confusion and build awareness for the need to address drowning.

The first statement—drowning prevention is not a synonym for water safety but is connected as both approaches share measures, and address common hazards, exposures, and vulnerabilities—was supported by 93.64% (n=103) second-round survey participants.

The second statement—when combined, drowning prevention and water safety includes measures that aim to prevent other water-related incidents and injuries, and promote water-related safe behaviours—was supported by 91.82% (n=101) of second-round survey participants.

The third statement—using ‘drowning prevention’ or ‘drowning prevention and water safety’ can reduce any confusion in contexts where water safety is associated with safe drinking WASH—was supported by 81.82% (n=90) of second-round survey participants.

## Discussion

Our study adopted a consensus-based approach to developing a conceptual definition for drowning prevention and aimed to strengthen the definition by addressing perceived confusion between the terms drowning prevention and water safety. The definition presents a starting point for framing and reframing drowning prevention in ways to inspire action, expand coalitions and strengthen institutions for multisectoral action.^3^

The concepts within our definition: (1) multidisciplinary approach, (2) evidence-informed measures, (3) reducing risk, (4) building resilience and (5) addressing hazards, exposure, vulnerability, provide a framework for understanding, developing and strengthening drowning prevention efforts. These concepts may aid the identification of cobenefits in sectors and agendas not yet engaged in drowning prevention and assist the development of interventions in ways not previously considered.

### Promoting multidisciplinary approaches

Multidisciplinary approaches combine academic and/or professional specialisations to solve a problem. Through the utilisation of a multidisciplinary approach, our definition emphasises the need to integrate diverse sectors and stakeholders each with their own contexts, expertise and approaches. Examples of multidisciplinary expertise engaged in drowning prevention include technical domains such as lifeguarding, emergency response management or education; academic fields such as epidemiology or engineering; management disciplines such as communications, advocacy, or administration, and regulators such as those in occupational health, maritime, and swimming pool safety. It is hoped that further synergistic efforts across disciplines can combine to increase comprehension, scope and efficiency of drowning prevention measures.

More than desirable, a multidisciplinary approach may be critical to the leadership and coordination of drowning prevention efforts. A review of drowning prevention planning shows that national leadership and coordination is not homogeneous, and varies across sectors and disciplines, in some examples including ministries of public health (Thailand), labour, invalids and social affairs (Viet Nam) and an alliance of non-government organisations (Australia)^10^ In some cases responsibility is fluid. For example, the Malaysian Water Activity Safety Committee was launched in 2015 under the Ministry for Local Government (discipline - public administration),^8^ and then moved to the Department of Fire and Rescue (discipline - emergency management) in 2022. Strengthening capacity for work in whole-of-society models, combining the resources, skills, and capabilities of each sector and stakeholder, whether government or non-government, is critical to successful multisectoral action and to advancing strategic priorities for drowning prevention.^10 12^

### Expanding evidence-informed measures

Our study presents the concept evidence-informed to describe drowning prevention measures, although there was some consideration of using evidence-based instead. Evidence-based practice has high relevance in medical settings where clinical expertise, and the best available research evidence is combined to make healthcare decisions.^22^ On the other hand evidence-informed practice combines research evidence, clinical experience, professional judgement, participant preferences and contextual factors to make decisions,^23^ and is designed to meet changing conditions with flexibility when evidence is weak or non-existent.^24^

The evidence base for drowning prevention is emergent, with many interventions commonly accepted by policy makers and practitioners considered to lack robust evidence.^25^ The challenge is that while these suggested interventions may lack the rigour of a scientific trial, they align with established best practices, and are often feasibility tested in the field. In this regard, the concept of evidence-informed aligns to the notion of drowning prevention as a multidisciplinary endeavour and necessitating the incorporation of contextual knowledge and methodologies from diverse sectors. A future research agenda should prioritise studies that explore greater contextualisation of drowning prevention, and measure the efficiency and efficacy of interventions.^12^

### Reframing drowning risk

Our definition reinforces the importance of drowning risk—the consequence of the interaction between a hazard, the characteristics that make people vulnerable to drowning, and their exposure (context, frequency of occurrence, duration) to a drowning hazard. The basis for this conceptualisation is used in disaster risk reduction where it underpins the Sendai Framework,^26^ and in the Intergovernmental Panel on Climate Change.^27^

Disaster risk reduction and climate resilience are two agendas prioritised for the integration of drowning prevention efforts.^12^ Reframing drowning prevention and aligning to the language and concepts used in disaster risk reduction and climate resilience sectors may assist that integration and help to facilitate the identification of cobenefits and complimentary interventions.

### Exploring hazard, vulnerability and exposure

The management of drowning risk more often focuses on mitigating hazards in aquatic environments. The consideration of exposure and vulnerability to drowning hazards may improve the assessment of risk and provide context essential to designing effective interventions.

A drowning hazard is an environmental or human induced feature or process that contributes to drowning risk. Drowning hazards can be (1) natural features for example, oceans, rivers, lakes and beaches, (2) natural processes including tropical storms, floods, tsunami, heatwaves, currents, waves steep or slippery banks or (3) human induced processes and features in the built environment or flaws in technological design, for example, modes of transportation, swimming pools, bathtubs and structurally deficient dams.

There is extensive research exploring the dangers of drowning hazards including rip currents^28^ and backyard swimming pools.^29^ Further research investigating the impacts of hydrometeorological hazards such as flooding,^30^ storm surge and heatwaves,^31^ on drowning risk is much needed.

Drowning exposure is the context, occurrence and duration in which an individual, community or population has contact with a specific drowning hazard. Exposure can be influenced by social, economic and environmental factors, and can occur in occupational, recreational or everyday living contexts. Calculating drowning risk based on exposure to hazards could supplement research based on population-based drowning estimates.^32^ Exposure is a concept important for monitoring adverse outcomes. A study found that learning to swim did not increase exposure or risk taking in Bangladeshi children.^33^ While exposure provides a basis for targeting interventions, high rates of exposure alone does not mean a person is at increased risk of drowning. For detailed assessment of drowning risk, exposure must be considered alongside vulnerability.

Drowning vulnerability is the susceptibility of an individual, community or population to drowning. Here, we use vulnerability as a dynamic concept that interacts with context, rather than as a label (vulnerable) applied to an individual or group.^34^ Vulnerability is best measured in layers and can be based on a range of physical, social, economic and environmental factors.

We caution against generalised use of the term vulnerable to apply to large cohorts without consideration of the individual traits that may contribute to vulnerability. Individual vulnerabilities include poor or insufficient swimming skills that might contribute to increased risk in a particular environment or water activity,^35^ medical conditions including heart disease,^36^ epilepsy^37^ and autism,^38^ and excessive alcohol or illicit drug use,^39^ which may increase susceptibility to drowning when a person is exposed to water.

Research investigating how social determinants increase exposure and vulnerabilities to drowning, including the impacts of where a person is born, the features of their neighbourhood and built environment in which they live, what access they have to education, safe working conditions and health services, is a high priority for the advancement of drowning prevention.^12^ A detailed analysis of the role of exposure and vulnerability may help in the targeting of prevention measures. For example, rock fisher drowning in Australia is an activity where participants are exposed to multiple hazards (rocks, waves, tidal changes), and where mortality data shows over representation of specific migrant populations—some of whom are thought to lack swimming skills, adding layers of vulnerability in the event of a fall into the wave zone.^32^ Studies have called for interventions that go beyond the delivery of translated messages, and address specific exposures and vulnerabilities of identified rock fisher populations.^32 40 41^ Those studies recommended direct advocacy via migrant community groups, provision of safety equipment to participants to reduce vulnerability in the event of a fall and increasing awareness of local weather forecasting when conditions are likely to increase exposure to hazards.

### Strengthening resilience for drowning prevention

Resilience is a concept used frequently in disaster risk reduction and health system contexts. Disaster resilience is the ability of a system, community or society to resist, absorb, accommodate and recover from the effects of a hazard in a timely and efficient manner.^26^ Health system resilience tends to focus on preparations for health shocks, by strengthening the policies, planning, health workforce and surveillance needed to minimise the consequences of such disruptions.^42^ Community resilience is described as a community’s intrinsic capacity to resist and recover from a disturbance, where communication, risk awareness, adaptation and risk governance are important elements.^43^ Community resilience is often measured across social, economic, institutional, physical and natural domains.^27^

In our definition, drowning prevention resilience means the adaptive and robust capacity of an individual, community or system to avoid, resist or respond to drowning risk, including pre and post event. For example, strengthening individual-level drowning prevention resilience includes the development of swimming, water safety and basic rescue skills in school children, parental education on supervision requirements around water, and public awareness of lifejacket laws. Building community-level drowning prevention resilience includes increasing the availability of designated and supervised places to swim, enhancing local beach lifeguard services and ensuring causeways have appropriate signage or gates in times of forecast flooding. Reinforcing system-level drowning prevention resilience includes upgrading national pool fencing legislation, monitoring safety standards for waterways and swimming pools, and strengthening of a drowning data surveillance system.

### Using the conceptual definition

The conceptual definition has many potential applications. Both researchers and safety practitioners can expand risk models to measure exposure, vulnerability, and hazards in calculating, reporting and addressing drowning risk. Policy makers can embrace multidisciplinary approaches in intervention planning and adopt whole-of-society coalitions for drowning prevention. Educators can reframe and redesign drowning prevention programmes to consider the concept of resilience. The use of the conceptual definition should be monitored in peer-review literature and grey documents. Adoption by WHO, the Global Alliance for Drowning Prevention,^12^ and international and national drowning prevention groups may increase use, and lead to further refinement and strengthening of the conceptual definition.

### Developing an operational definition

There may be utility in operationalising the definition for use in research and evaluation. An operational definition sets indicators to measure the degree to which the definition threshold is achieved. While not an objective of this study, indicators in an operational definition could include the breakdown of hazards, exposures and vulnerabilities into variables. Such a framework could enhance evaluation. For example, a programme or policy targeting a hazard without considering the role of exposure, or vulnerability could be improved if all three factors were addressed.

### Limitations

Our document review may have missed relevant studies not specifically mentioning ‘drowning’ in title, abstracts or key words. A more comprehensive search of grey literature and non-indexed journals may have identified additional data relevant to defining drowning prevention.

Our recruitment methods are a strength and a weakness. We sought to recruit participants that reflected diverse expertise, as studies show drowning prevention is not homogeneous, and involves various sectors.^3 10^ It is possible that the definition, while developed by consensus-based agreement, does not completely resonate with a specific sector. For example, leisure and education sector practitioners might place greater emphasis on individual knowledge or skill development.

The Delphi method captured widespread input, however, the process of anonymising feedback meant some points raised by participants could not easily be explored or progressed to develop deeper insight. Anonymising participants meant we were unable to identify representation from specific organisations, governments or academic centres for follow-up.

Our study was conducted in English, which may have impacted on non-native English speaker participation. Although they completed the surveys, 24.6% of our participants indicated that English was not their preferred language. Preferred languages included Bangla, (4.6%) French (3.7%), a Chinese language (3.0%), Portuguese (3.0%) or Spanish (3.0%). Translating and evaluating the definition in languages other than English may be beneficial.

## Conclusion

Using the Delphi method enabled the codevelopment of the conceptual definition for drowning prevention. The Delphi method provided for diverse input that may have not been possible had other methods been employed. By embedding the concepts of multidisciplinary, evidence-informed, and drowning risk, hazard, vulnerability, exposure and resilience, the definition seeks to enhance the universality of approaches to multisectoral action, and support efforts to partner with other health and sustainable development agendas. The definition provides clear guidance to governments, advocates, researchers, practitioners and funders, and aims to reduce the risks associated with the application of disparate or heterogeneous concepts of drowning prevention.

## Data Availability

All data relevant to the study are included in the article. All data relevant to the study are included in the article or uploaded as online supplemental information.
