# Supplementary material for: Conceptual definition for drowning prevention: a Delphi study
Source: Inj Prev. 2023 Nov 9;30(2):145–52. doi: 10.1136/ip-2023-045085 (PMC10958290; doi:10.1136/ip-2023-045085)
Supplement: Supplementary data [file ip-2023-045085supp001.pdf]

SUPPLEMENT

Drowning prevention: a conceptual definition using Delphi-method

Authors

Mr. Justin-Paul Scarr, MBA; Royal Life Saving Society—Australia, Sydney, Australia and The George Institute for Global Health, University of New South Wales, Sydney, Australia.

Dr Jagnoor Jagnoor, PhD; The George Institute for Global Health, New Delhi, India and The George Institute for Global Health, University of New South Wales, Sydney, Australia.

Corresponding Author

Mr. Justin-Paul Scarr

Royal Life Saving Society—Australia, PO Box 558, Broadway, NSW, Australia

The George Institute for Global Health, University of New South Wales, Level 5, 1 King Street, Newtown 2042 NSW, Australia

Email : jscarr@georgeinstitute.org.au

Phone : +61 408 434 011

Supplement 1:

Survey 1 questions constructing the definition.

BEFORE STARTING ON THE PRIORITIES, LETS FOCUS ON WHAT DROWNING PREVENTION MEANS TO YOU.

Achieving a common understanding of drowning prevention is important to setting priorities. Our scoping review found no agreed definition for drowning prevention, and some variations in how the term is used. (Link)

CONSTRUCTING A DEFINITION OF DROWNING PREVENTION

Here we present a structural approach to drafting a definition for drowning prevention. Our aim is to construct, review and build consensus for a definition. Once completed, the final field presents your constructed definition for review. And then you are ready to move to the priorities.

Drowning prevention is (insert)	○ an approach that<br>○ a public health approach that<br>○ a multidisciplinary approach that<br>○ a concept or practice that<br>○ any action that
aims to (insert)<br><br>(multiple choices allowed here)	☐ reduce risk and build resilience<br>☐ address drowning hazards, exposures and vulnerabilities<br>☐ implement measures
to protect (insert)	○ a person<br>○ people<br>○ an individual, community or population
against (insert)	○ submersion<br>○ fatal and non-fatal submersion<br>○ fatal and non-fatal submersion, and other injuries<br>○ drowning
in (insert)	○ a body of water<br>○ on or around a body of water<br>○ a liquid

Based on your responses above the structured definiton for drowning prevention is:

Drowning prevention is [drowning_is] aims to [aims_to_insert] to protect [to_protect_insert] against [against_insert] in [in_insert].

If you have any further suggestions for this definition, please add them here. ______________________

15/08/2023 8:55am projectredcap.org REDCap
